# Supplementary material for: Upper airway gene expression reveals suppressed immune responses to SARS-CoV-2 compared with other respiratory viruses
Source: Nat Commun. 2020 Nov 17;11:5854. doi: 10.1038/s41467-020-19587-y (PMC7673985; doi:10.1038/s41467-020-19587-y)
Supplement: Supplementary file 1 — Supplementary Information [file 41467_2020_19587_MOESM1_ESM.pdf]

Supplementary Tables

Supplementary Table 1. Cohort clinical and demographic characteristics.

	Cohort Overall		COVID-19	Other Viral ARI	Non-Viral ARI
Total Enrolled	234		93	41	100
*Age, years (mean, range)	52 (20 - 85+)		47	51	57
Female gender	124	53%	50	19	55
**Clinical Encounter Type**	n	%	n	n	n
Inpatient	47	20%	4	9	34
Intensive Care Unit	19	8%	4	6	9
Emergency Department	41	18%	5	12	24
Outpatient	92	39%	52	14	26
Unknown	35	15%	28	0	7
**Race**	n	%	n	n	n
White or Caucasian	93	40%	19	27	47
Asian	43	18%	13	10	20
Black or African American	20	9%	3	1	16
Native Hawaiian	1	0%	1	0	0
Other	39	17%	27	3	9
Unknown	38	16%	30	0	8
**Ethnicity**	n	%	n	n	n
Not Hispanic or Latino	159	68%	40	39	80
Hispanic or Latino	33	14%	21	1	11
Other/Unknown	42	18%	32	1	9
**Sample Type**	n	%	n	n	n
NP Swab	112	48%	45	24	43
Pooled NP+OP Swab	86	37%	19	17	50
Unknown	36	15%	29	0	7

ARI = Acute Respiratory Illness, NP = Nasopharyngeal, OP = Oropharyngeal.
*Available for 223 subjects (95%)

Supplementary Table 2.

a. Performance of classifier models measured by mean (range) AUROC in 5-fold cross-validation.

Model	COVID-19 vs. All Other ARI	COVID-19 vs. Non-Viral ARI	COVID-19 vs. Other Viral ARI
27-gene	0.981 (0.955-0.994)	0.987 (0.968-1.000)	0.966 (0.921-1.000)
+age/gender	0.974 (0.949-0.994)	0.983 (0.963-1.000)	0.952 (0.914-0.980)
10-gene	0.954 (0.932-0.962)	0.971 (0.958-0.989)	0.912 (0.868-0.947)
3-gene	0.885 (0.852-0.935)	0.932 (0.889-0.984)	0.767 (0.646-0.936)

b. Accuracy, sensitivity, specificity, positive predictive value (PPV) and negative predictive value (NPV) of sparse classifier models, assuming an 18% (41/234) prevalence of other viral ARIs, at different cutoff thresholds for out-of-fold predicted probability.

Model	Threshold	Accuracy	Sensitivity	Specificity	PPV	NPV
27-gene	0.5	0.923	0.882	0.95	0.921	0.924
10-gene	0.5	0.872	0.839	0.894	0.839	0.894
3-gene	0.5	0.833	0.763	0.879	0.807	0.849
27-gene	0.4	0.923	0.957	0.901	0.864	0.969
10-gene	0.4	0.88	0.882	0.879	0.828	0.919
3-gene	0.4	0.838	0.806	0.858	0.789	0.871

Supplementary Table 3. Lasso-selected features and coefficients of classifier models.

27-gene model	
(Intercept)	-2.834
CRLF1	-0.183
TRO	0.255
PCSK5	0.016
TIMP1	-0.277
ICAM4	-0.15
IFI6	0.742
WDR74	0.216
TNS3	-0.106
IFI44L	0.061
PLK4	0.01
FAM83A	0.082
ADM	-0.141
PPEF2	0.033
DGKI	0.066
SCGB3A1	-0.074
KLF15	-0.038
KRT13	-0.107
RGPD2	-0.194
DCUN1D3	-0.168
BPIFA1	0.005
MUC2	-0.012
MUC19	0.037
EIF3CL	-0.029
HBA1	-0.038
IGLL5	0.103
AL928654.3	-0.086
SPECC1L-ADORA2A	-0.079

10-gene model	
(Intercept)	-4.733
PCSK5	0.046
IL1R2	-0.055
IL1B	-0.041
IFI6	0.452
WDR74	0.124
FAM83A	0.004
ADM	-0.099
IFI27	0.084
KRT13	-0.005
DCUN1D3	-0.05

3-gene model	
(Intercept)	-2.808
IL1R2	-0.037
IL1B	-0.052
IFI6	0.372

Supplementary Figures

Supplementary Figure 1. Cohort Characteristics.

a Prevalence of other pathogenic respiratory viruses identified by mNGS in study subjects. Two patients had co-infections with both RSV and HRV. CoV, Coronavirus; HRV, Human Rhinovirus; Flu, Influenza Virus; HMPV, Human Metapneumovirus; RSV, Respiratory Syncytial Virus; PIV, Parainfluenza Virus. **b** Correlation of SARS-CoV-2 PCR Crossing Threshold (Ct) and mNGS reads-per-million (rpM; $\log_2$ scale) for n=92 patients in the SARS-CoV-2 group with rpM > 0. Ct represents an average across the SARS-CoV-2 genomic loci assessed.

Supplementary Figure 2. Distinctions and Commonalities in the Host Response between SARS-CoV-2 and Other Respiratory Viruses.

a Scatter plots of normalized gene counts ($\log_2$ scale, y-axis) as a function of SARS-CoV-2 viral load ($\log_{10}$(rpM), x-axis) for the most significant interferon response genes induced by SARS-CoV-2, and the SARS-CoV-2 receptor gene *ACE2*. Robust regression was performed on SARS-CoV-2 positive patients with $\log_{10}$(rpM)>=0 (n=82) to characterize the relationship to viral load. Shaded bands represent 95% confidence intervals. Statistical results listed for each gene refer to, from top to bottom: the regression analysis (p-values for difference of the slope from 0 derived from a t-statistic and Benjamini-Hochberg adjusted; R^2 is the adjusted robust coefficient of determination), the DE analysis between SARS-CoV-2 (n=93) and non-viral ARIs (n=100; p-values derived from a moderated t-statistic and Benjamini-Hochberg adjusted), and the DE analysis between SARS-CoV-2 and other viral ARIs (n=41; p-values derived from a moderated t-statistic and Benjamini-Hochberg adjusted). FC, fold-change. **b** Gene set enrichment analysis for the direct comparison between patients with SARS-CoV-2 and other viral ARIs. Pathway p-values were calculated using an adaptive, multilevel splitting Monte Carlo approach and Benjamini-Hochberg adjusted. **c** Scatter plots of normalized gene counts ($\log_2$ scale, y-axis) as a function of SARS-CoV-2 viral load ($\log_{10}$(rpM), x-axis) for selected interferon response genes in the leading edge of the interferon signaling gene set, showing lagging expression in SARS-CoV-2 compared to other viral ARIs. Robust regression was performed on SARS-CoV-2 positive patients with $\log_{10}$(rpM)>=0 (n=82) to characterize the relationship to viral load. Shaded bands represent 95% confidence intervals. Statistical results listed for each gene as in a. FC, fold-change.

Supplementary Figure 3. Expression Differences of Genes of Interest across Different Respiratory Virus Types.

Boxplots of quantile normalized gene counts ($\log_2$ scale) across patient groups, with other viral ARIs broken down by virus type (two patients with viral co-infections were assigned to the higher abundance virus). The horizontal line inside the box denotes the median. The lower and upper hinges correspond to the first and third quartiles, respectively. Whiskers extend from the hinge to the largest (smallest, respectively) value no more than 1.5*IQR away from the hinge, where IQR is the inter-quartile range. Two-sided Mann-Whitney-Wilcoxon tests were used to compare virus types with sufficient sample size to SARS-CoV-2, followed by Bonferroni's correction. **a** Interferon response genes. **b** IL-1 and inflammasome pathway genes. **c** Genes increased in expression in SARS-CoV-2 compared to both non-viral and other viral ARIs. No, no virus (n=100); SC2, SARS-CoV-2 (n=93); HRV, Human Rhinovirus (n=13); Flu, Influenza Virus (n=11); CoV, Coronavirus (other than SARS-CoV-2), n=7; HMPV, Human Metapneumovirus (n=6); RSV, Respiratory Syncytial Virus (n=3); PIV, Parainfluenza Virus (n=1).

Supplementary Figure 4. Full Cell Type Proportions Analysis.

In silico estimation of cell type proportions in the bulk RNA-sequencing using lung single cell signatures. The horizontal line inside the box denotes the median. The lower and upper hinges correspond to the first and third quartiles, respectively. Whiskers extend from the hinge to the largest (smallest, respectively) value no more than 1.5*IQR away from the hinge, where IQR is the inter-quartile range. The y-axis in each panel was trimmed at the maximum value among the three patient groups of 1.5*IQR above the third quartile. Pairwise comparisons between patient groups were performed with a two-sided Mann-Whitney-Wilcoxon test followed by Bonferroni's correction (n=93 SARS-CoV-2, n=41 other virus, n=100 no virus).

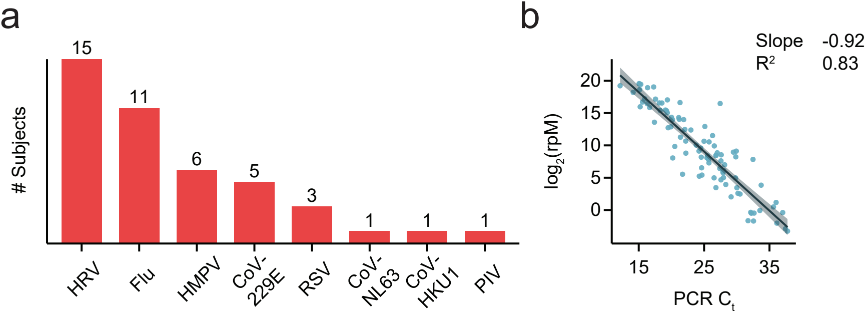

Supplementary Figure 1

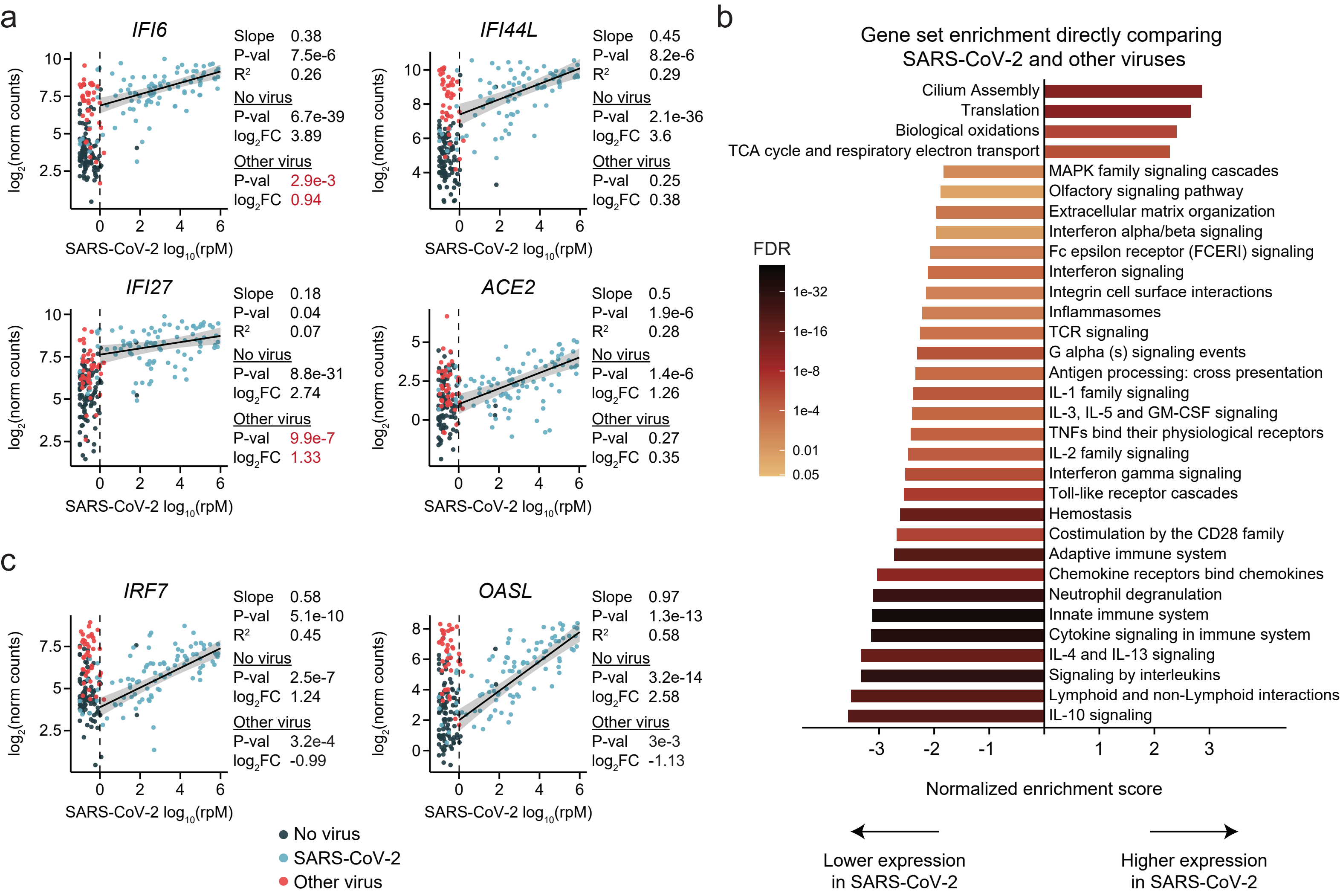

Supplementary Figure 2

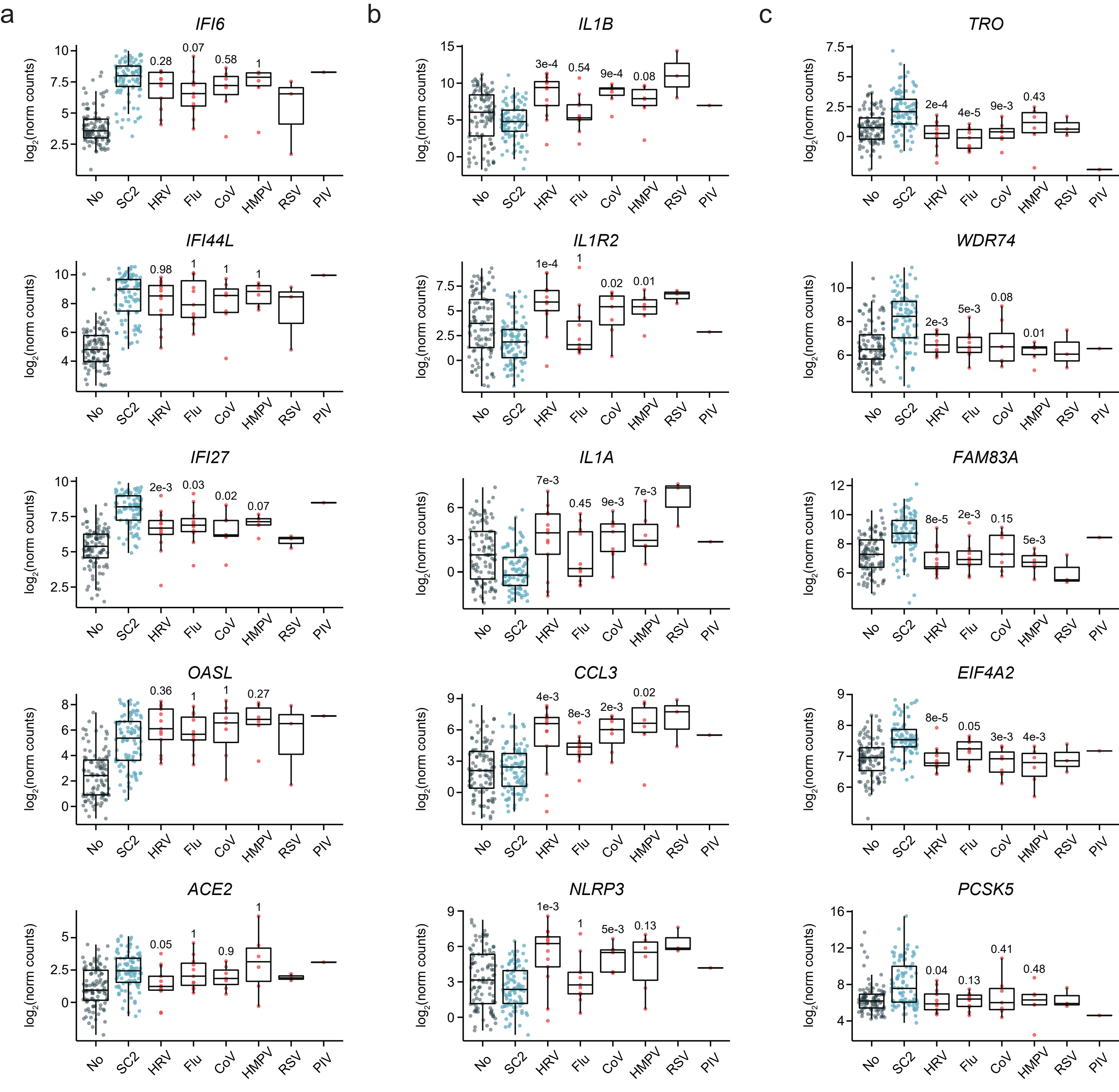

Supplementary Figure 3

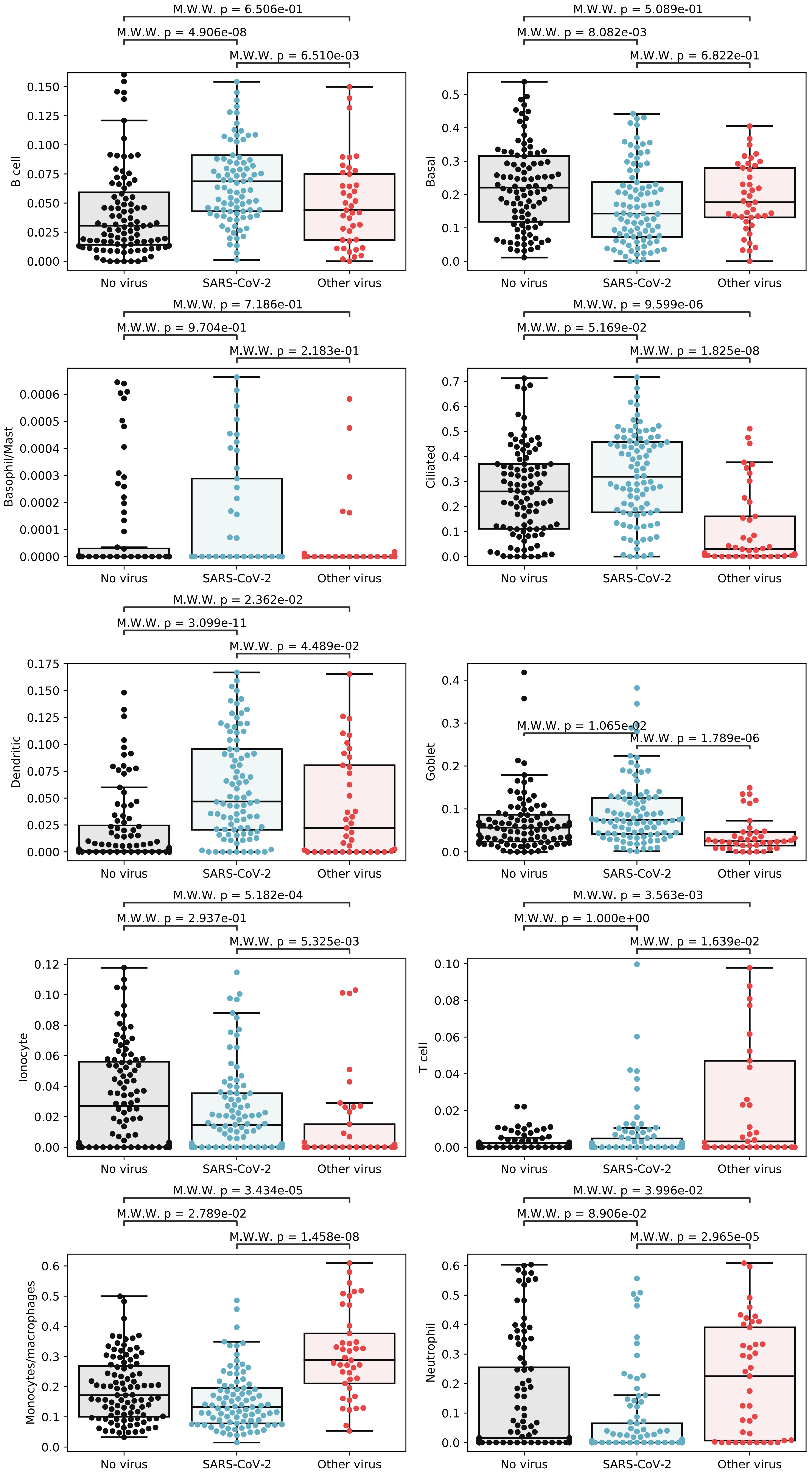

M.W.W. p = 6.506e-01
M.W.W. p = 4.906e-08
M.W.W. p = 6.510e-03
B cell
No virus
SARS-CoV-2
Other virus
M.W.W. p = 5.089e-01
M.W.W. p = 8.082e-03
M.W.W. p = 6.822e-01
Basal
No virus
SARS-CoV-2
Other virus
M.W.W. p = 7.186e-01
M.W.W. p = 9.704e-01
M.W.W. p = 2.183e-01
Basophil/Mast
No virus
SARS-CoV-2
Other virus
M.W.W. p = 9.599e-06
M.W.W. p = 5.169e-02
M.W.W. p = 1.825e-08
Ciliated
No virus
SARS-CoV-2
Other virus
M.W.W. p = 2.362e-02
M.W.W. p = 3.099e-11
M.W.W. p = 4.489e-02
Dendritic
No virus
SARS-CoV-2
Other virus
M.W.W. p = 1.065e-02
M.W.W. p = 1.789e-06
Goblet
No virus
SARS-CoV-2
Other virus
M.W.W. p = 5.182e-04
M.W.W. p = 2.937e-01
M.W.W. p = 5.325e-03
Ionocyte
No virus
SARS-CoV-2
Other virus
M.W.W. p = 3.563e-03
M.W.W. p = 1.000e+00
M.W.W. p = 1.639e-02
T cell
No virus
SARS-CoV-2
Other virus
M.W.W. p = 3.434e-05
M.W.W. p = 2.789e-02
M.W.W. p = 1.458e-08
Monocytes/macrophages
No virus
SARS-CoV-2
Other virus
M.W.W. p = 3.996e-02
M.W.W. p = 8.906e-02
M.W.W. p = 2.965e-05
Neutrophil
No virus
SARS-CoV-2
Other virus
