# Supplementary material for: Upper airway gene expression reveals suppressed immune responses to SARS-CoV-2 compared with other respiratory viruses
Source: Nat Commun. 2020 Nov 17;11:5854. doi: 10.1038/s41467-020-19587-y (PMC7673985; doi:10.1038/s41467-020-19587-y)
Supplement: Supplementary file 3 — Description of Additional Supplementary Files [file 41467_2020_19587_MOESM3_ESM.pdf]

Description of Additional Supplementary Files

File Name: Supplementary Data 1
Description: Clinical diagnoses for patients with no virus detected.

File Name: Supplementary Data 2
Description: Differential expression and regression analyses.

File Name: Supplementary Data 3
Description: Gene set enrichment analyses.

File Name: Supplementary Data 4
Description: Cell type proportions analysis.

File Name: Supplementary Data 5
Description: Sequencing statistics per sample.
