# Supplementary material for: Upper airway gene expression reveals suppressed immune responses to SARS-CoV-2 compared with other respiratory viruses
Source: Nat Commun. 2020 Nov 17;11:5854. doi: 10.1038/s41467-020-19587-y (PMC7673985; doi:10.1038/s41467-020-19587-y)
Supplement: Supplementary file 9 — Reporting Summary [file 41467_2020_19587_MOESM9_ESM.pdf]

nature research

Corresponding author(s): Langelier, Charles

Last updated by author(s): Oct 9, 2020

Reporting Summary

Nature Research wishes to improve the reproducibility of the work that we publish. This form provides structure for consistency and transparency in reporting. For further information on Nature Research policies, see our Editorial Policies and the Editorial Policy Checklist.

Statistics

For all statistical analyses, confirm that the following items are present in the figure legend, table legend, main text, or Methods section.

n/a	Confirmed	
☐	☒	The exact sample size (*n*) for each experimental group/condition, given as a discrete number and unit of measurement
☐	☒	A statement on whether measurements were taken from distinct samples or whether the same sample was measured repeatedly
☐	☒	The statistical test(s) used AND whether they are one- or two-sided<br>*Only common tests should be described solely by name; describe more complex techniques in the Methods section.*
☐	☒	A description of all covariates tested
☐	☒	A description of any assumptions or corrections, such as tests of normality and adjustment for multiple comparisons
☐	☒	A full description of the statistical parameters including central tendency (e.g. means) or other basic estimates (e.g. regression coefficient) AND variation (e.g. standard deviation) or associated estimates of uncertainty (e.g. confidence intervals)
☐	☒	For null hypothesis testing, the test statistic (e.g. F, t, r) with confidence intervals, effect sizes, degrees of freedom and P value noted<br>*Give P values as exact values whenever suitable.*
☒	☐	For Bayesian analysis, information on the choice of priors and Markov chain Monte Carlo settings
☒	☐	For hierarchical and complex designs, identification of the appropriate level for tests and full reporting of outcomes
☒	☐	Estimates of effect sizes (e.g. Cohen's *d*, Pearson's *r*), indicating how they were calculated

Our web collection on statistics for biologists contains articles on many of the points above.

Software and code

Policy information about availability of computer code

Data collection

Data was collected using RNA sequencing of nasopharyngeal swab samples from patients under investigation for COVID-19. Libraries were prepared using a modified metagenomic spiked sequencing primer enrichment (MSSPE) method and sequenced on an Illumina NovaSeq 6000 instrument, generating 2x146bp paired-end reads. Sequencing reads were pseuo-aligned to transcripts of human protein coding genes with kallisto (v. 0.46.1) (Bray et al. 2016) and gene-level counts were generated using the R package tximport (v. 1.14).

Data analysis

Human gene counts data was analyzed using the following tools/packages:
1. Variance stabilizing transformation was performed using the R package DESeq2 (v. 1.26).
2. Differential expression (DE) analyses were performed using the R package limma (v. 3.42).
3. Gene set enrichment analyses were performed using the R package fgsea (v. 1.13.5).
4. Robust regression of gene counts on viral load was performed using the R packages robustbase (v. 0.93.6) and ggeffects (v. 0.14.3).
5. Gene expression classifiers were constructed using the R packages glmnet (v. 4.0-2) and randomForest (v. 4.6-14).
6. Cell type proportions analysis was performed using the CIBERSORT X algorithm (Newman et al. 2015).

Metagenomic analysis was performed using the IDSeq pipeline (v. 4.3), kraken2 (v. 2.0.8_beta), minimap2 (v. 2.17) and the R package MASS (v. 7.3-51).

Code for the DE analyses, cell type proportions analysis and gene expression classifiers are available at:
https://github.com/czbiohub/covid19-transcriptomics-pathogenesis-diagnostics-results

For manuscripts utilizing custom algorithms or software that are central to the research but not yet described in published literature, software must be made available to editors and reviewers. We strongly encourage code deposition in a community repository (e.g. GitHub). See the Nature Research guidelines for submitting code & software for further information.

Data

Policy information about availability of data

All manuscripts must include a data availability statement. This statement should provide the following information, where applicable:

- Accession codes, unique identifiers, or web links for publicly available datasets
- A list of figures that have associated raw data
- A description of any restrictions on data availability

Human gene counts and metadata for the samples generated in this study can be obtained at: https://github.com/czbiohub/covid19-transcriptomics-pathogenesis-diagnostics-results. Gene counts have also been deposited under NCBI GEO accession GSE156063 [https://www.ncbi.nlm.nih.gov/geo/query/acc.cgi?acc=GSE156063]. IDSeq metagenomic analysis results are available at https://idseq.net/ under project name "covid19_transcriptomics_pathogenesis_diagnostics". Raw mNGS FASTQ files, subtracted of human-mapping reads for privacy reasons, are available under NCBI BioProject accession PRJNA633853 [https://www.ncbi.nlm.nih.gov/bioproject/?term=PRJNA633853]. The independent NP swab mNGS dataset we re-analyzed can be obtained according to the Data Availability statement in the original publication (Ramlall et al. 2020). The published human lung single-cell datasets (Travaglini et al. 2020) used for cell type proportions analysis can be obtained through Synapse under accessions syn21560510 and syn21560511.

Field-specific reporting

Please select the one below that is the best fit for your research. If you are not sure, read the appropriate sections before making your selection.

- [x] Life sciences
- [ ] Behavioural & social sciences
- [ ] Ecological, evolutionary & environmental sciences

For a reference copy of the document with all sections, see nature.com/documents/nr-reporting-summary-flat.pdf

Life sciences study design

All studies must disclose on these points even when the disclosure is negative.

Sample size	Initial sample size estimates were based on learning curve analyses from a prior transcriptional profiling study of respiratory tract infections (Langelier et al. 2018). These analyses suggested that a sample size of > 20 subjects in each comparator group (COVID-19, other viral, and non-viral acute respiratory illness) would be sufficient to yield an AUC of 95%.
Data exclusions	We excluded from analysis a small number of samples that did not neatly fit into our 3 patient groups (COVID-19, other viral, non-viral acute respiratory illness). Specifically, 3 patients in the non-viral group who had a positive viral test of some kind in their medical record within 7 days of the sample collected for this study, and 1 patient in the COVID-19 group that had a co-infection with another respiratory virus detected by mNGS.<br>We also excluded samples with less than 400,000 estimated counts associated with transcripts of protein coding genes since we judged it was not possible to reliably assess gene expression in these samples in comparison to more deeply sequenced samples.
Replication	Our key findings in the differential expression analysis between COVID-19 and other viral infections were validated in a re-analysis we performed of a published, independent dataset of NP swab mNGS sequencing.<br>To verify reproducibility of the classifier findings, we used 5-fold cross-validation. For each train-test split, we used a nested cross-validation within the training set to select the lasso tuning parameter.
Randomization	No randomization was involved in this observational study. Participants a-priori belonged to groups based on acute respiratory illness (ARI) etiology: COVID-19, other viral ARI, non-viral ARI.
Blinding	Blinding was not relevant since the goal of the study was to determine observational differences between pre-defined groups of patients, and it did not involve any treatment/experiment.

Reporting for specific materials, systems and methods

We require information from authors about some types of materials, experimental systems and methods used in many studies. Here, indicate whether each material, system or method listed is relevant to your study. If you are not sure if a list item applies to your research, read the appropriate section before selecting a response.

Materials & experimental systems

n/a	Involved in the study	
[x]	[ ]	Antibodies
[x]	[ ]	Eukaryotic cell lines
[x]	[ ]	Palaeontology and archaeology
[x]	[ ]	Animals and other organisms
[ ]	[x]	Human research participants
[x]	[ ]	Clinical data
[x]	[ ]	Dual use research of concern

Methods

n/a	Involved in the study	
[x]	[ ]	ChIP-seq
[x]	[ ]	Flow cytometry
[x]	[ ]	MRI-based neuroimaging

Human research participants

Policy information about studies involving human research participants

Population characteristics	Patients with acute respiratory illness under investigation for COVID-19 and over the age of 18. No limitations based on gender, race/ethnicity, genetic factors or setting of care. Full cohort characteristics are available in Supp. Table 1.
Recruitment	We conducted an observational cohort study of 234 patients with acute respiratory illnesses tested for COVID-19 at the University of California, San Francisco (UCSF) and Zuckerberg San Francisco General Hospital. We evaluated leftover RNA extracted from clinical swab specimens processed at the UCSF Clinical Microbiology Laboratory. The UCSF Institutional Review Board granted a waiver of consent for this study, which was part of a larger ongoing surveillance study of patients with outbreak-associated viral and bacterial infections (UCSF IRB protocol 17-24056).<br>Inclusion criteria were: 1) status as a patient under investigation for COVID-19, 2) age of 18 years or older, 3) a clinician-ordered test for SARS-CoV-2 was performed between 03/10/2020 and 04/07/2020 using reverse transcription polymerase chain reaction (RT-PCR) from a nasopharyngeal (NP) swab, obtained with or without an oropharyngeal (OP) swab, and 4) excess extracted RNA was available for metagenomic sequencing. If more than one sample was collected from a patient ultimately diagnosed with COVID-19, only the first available positive sample was analyzed. Demographic and clinical characteristics were assessed exclusively from each institution's Epic-based electronic health record.
Ethics oversight	University of California, San Francisco IRB protocol 17-24056

Note that full information on the approval of the study protocol must also be provided in the manuscript.
